# Motives for adult participation in physical activity: type of activity, age, and gender

**DOI:** 10.1186/s12889-015-1429-7

**Published:** 2015-01-31

**Authors:** Keyvan Molanorouzi, Selina Khoo, Tony Morris

**Affiliations:** Sports Centre, University of Malaya, Kuala Lumpur, 50603 Malaysia; Institute of Sport, Exercise and Active Living (ISEAL), College of Sport and Exercise Science, Victoria University, Melbourne, Australia

**Keywords:** Malaysia, Construct validity, Physical Activity and Leisure Motivation Scale (PALMS), Discriminant function analysis

## Abstract

**Background:**

In recent years, there has been a decline in physical activity among adults. Motivation has been shown to be a crucial factor in maintaining physical activity. The purpose of this study was to examine whether motives for participation could accurately discriminate gender, age, and type of physical activity.

**Methods:**

A quantitative, cross-sectional descriptive research design was employed. The Physical Activity and Leisure Motivation Scale (PALMS) was used to assess motives for physical activity in 1,360 adults (703 males, 657 females) who had been exercising regularly for at least six months. The PALMS consists of 40 items that constitute eight sub-scales (mastery, enjoyment, psychological condition, physical condition, appearance, others’ expectations, affiliation, competition/ego). Respondents were divided into two age groups (young adults aged 20 to 40 years and middle-aged adults 41 to 64 years) and five types of activity (individual racing sports plus bowls, team sports, racquet sports, martial arts, and exercise).

**Results:**

The group discriminant function analyses revealed significant canonical functions correctly classifying the cases into gender (82%), age group (83%), team sport players 76%, individual racing sport plus bowls players 91%, racquet sport players 90%, exercisers 84%, and martial art players 91%. The competition/ego, appearance, physical condition, and mastery sub-scales contributed most to gender differences. Five sub-scales (mastery, psychological condition, others’ expectations, affiliation, and enjoyment) contributed most to the discriminant function for age. For type of activity, different sub-scales were the strongest contributors to the discriminant function for each type of PA.

**Conclusion:**

The findings in this study suggest that strong and important motives for participation in physical activity are different across type of activity, age, and gender in adults. Understanding the motives that influence physical activity participation is critical for developing interventions to promote higher levels of involvement.

## Background

The link between regular physical activity (PA) and physical and psychological health has been well documented in the literature [1,2]. The most important benefits of regular PA include reduced prevalence of many diseases, as well as decreased mortality [3-6]. Indeed, individuals of all ages can gain an array of physical, psychological, social, and emotional benefits from PA [7,8].

Physical inactivity is the fourth leading risk factor for global mortality and estimated to cause 6% of deaths worldwide [9]. An estimated 30% of the global ischemic heart disease burden, 27% of diabetes, and 21% of breast and colon cancer burden are attributable to physical inactivity. In Malaysia, the National Health and Morbidity Survey [10] reported that 35.7% of adults surveyed were physically inactive, with 30.5% men and 41% women classified as inactive, based on a combination of walking, moderate-intensity or vigorous-intensity activities achieving a minimum of at least 600 MET-minutes/week indicated by the IPAQ questionnaire.

Despite the established physical and mental health benefits of regular PA, a large proportion of the population in the United States [11], Europe [12] and Malaysia [13] do not participate in adequate PA to gain these health benefits, either being not sufficiently active or maintaining a sedentary lifestyle. For instance, it was found that the majority of Malaysian adults had low (35%) and moderate (62%) PA levels [13].

Increasing levels of PA to meet current guidelines during adulthood is a public health priority and PA recommendations and guidelines have been designed by public health professional organizations [9]. Therefore, identifying factors associated with adult PA behavior is important because it will help in informing future research and may guide the implementation of interventions aimed at promoting PA behavior across the lifespan. It is particularly important to identify and promote those factors that lead to sustained PA in the long term.

For these reasons, researchers, health professionals, and policy makers have all sought to explore the reasons why some people are physically active, whereas others are not. Although the antecedents to participation in PA are highly complex [14], one promising approach is to focus on motivation because it is a key factor that influences individuals’ initiation and maintenance of behavior [15]. Motivation not only affects PA participation, but is also a critical factor in exercise adherence [16-18]. Based on self-determination theory (SDT) motivation to engage in PA can include intrinsic and/or extrinsic aspects. Intrinsic motivation refers to engaging in an activity for the pleasure and inherent satisfaction [19]. Intrinsically-motivated individuals experience choice in their behavioral dispositions and an optimum level of challenge, thereby fulfilling their needs for autonomy and competence. For instance, a basketball player who trains regularly for the inherent fun and challenge involved in the game is said to be intrinsically motivated. On the other hand, extrinsic motivation refers to engaging in an activity for instrumental reasons, such as external pressures or rewards. Extrinsically-motivated individuals experience little optimal challenge or autonomy. For example, an athlete who competes in a sport because of the pressures from the coach or need for status or approval from family or friends is said to be extrinsically motivated [19,20]. Researchers have reported that extrinsic motives are critical during the early steps of PA adoption, whereas intrinsic motives are key for the maintenance of PA programmes [21,22].

Research on participation motivation suggests that there are systematic differences between participation motives and some demographic variables. These include gender [23], age [24,25], country [26,27], and preference for specific forms of PA [21,28]. Research on gender differences in participation motivation indicates that males and females exhibit different motives for participation in PA. Egli et al. [23] found that male college students were motivated most by what Egli et al. termed intrinsic factors (strength, competition, and challenge), whereas females were motivated by extrinsic factors (weight management and appearance). In another study, Chowdhury [29] found similar differences between the motives reported by males and females. Male adults reported higher motivation for affiliation and challenge, whereas female adults reported higher motivation for appearance. Also, Morris, Clayton, Power and Han [30] examined motives for participation in physical activity by gender, using discriminant function analysis. The strongest discriminating factors for gender were found to be health, challenge and status. Health was rated higher by females than males, and challenge and status were found to be more important for males than females.

In the adult population, it is well established that PA participation decreases significantly as age increases [10,31,32] and motivation for PA may also vary with age [30,33]. For example, middle-aged adults were found to be less intrinsically motivated compared to younger adults [24]. Trujillo, Brougham, and Walsh [33] tested the hypothesis that there are age-related differences in reasons for exercising. Participants included 212 people aged 18 to 34 years, 107 individuals aged 35 to 51 years, 79 people aged 52 to 68 years, and 59 individuals aged 69 to 86 years. Results provided mixed support for the hypotheses that younger individuals exhibit greater concern for interpersonal attraction outcomes, whereas older individuals exhibit greater concern for health outcomes. Based on developmental theories that focus on age-related tasks young adults are concerned with establishing intimate relationships [34-36]. Maintaining or improving physical appearance motivates younger adults to be physical activity, because physical appearance is an important component in attracting a partner in American society and many other cultures. Older adults are involved with evaluating their lives and searching for meaning. Erikson [36] suggested that the outcome of these evaluations determines if older adults will avoid despair and depression. Given that this evaluation process is a psychological task, we predict that older participants will exhibit more concern for consequences related to psychological health than younger adults. This hypothesis is consistent with research indicating that active older adults were motivated to exercise by the recognition they received from important others [33].

Very limited research has specifically examined motivation for certain types of PA. It is plausible that there is a relationship between motives for participation in PA and the types of PA that individuals choose to spend their time doing. Studies that have reported the correspondence of participation motives with specific types of PA suggest systematic differences [21,28,30,37]. For example, Frederick and Ryan [38] compared the motivation of those who participated in individual sports (such as tennis and sailing) and those who participated in fitness or exercise-oriented activities (such as running and aerobics), using the Motives for Physical Activity Measure (MPAM), which they developed in that study. They found that those who participated in individual sports had higher interest/enjoyment and competence motivation, whereas those who participated in fitness or exercise activities had higher body-related motivation. In one of the few studies on PA motivation, Morris et al. [30] examined five types of activity for PA participation: team sports (lacrosse, netball, basketball, volleyball), individual sports (gymnastics, swimming), racquet sports (tennis, table tennis, squash), exercise activities (aerobics, weight training), and martial arts (karate, tae kwon do, taiji). They measured motives for participation using a modified version of the Participation Motivation Questionnaire (PMQ) [39]. Morris et al. applied Discriminant Function Analysis (DFA) to determine the motives that distinguished each type of activity from the rest of the sample. Results showed that team sport participants were discriminated from all the other participants by higher scores on the social or affiliation sub-scale of the PMQ. Racquet sport competitors were discriminated by higher scores than the rest of the sample on the challenge or competition/ego sub-scale. Exercise participants were discriminated by higher scores on physical condition than participants in other activities. Morris et al. also found that martial arts competitors were discriminated by higher scores on enhancing body and mind-related skills than the rest of the sample.

Individuals vary from one to another; some like to exercise alone, whereas others like to be in a group. Some individuals like their PA program to be tailored, whereas others prefer some level of personal choice. PA advice, particularly in primary health care, may be an effective tool to promote active lifestyles at the population level [40]. According to SDT, long-term adherence to PA could be improved by designing programmes or interventions that target the more autonomous reasons for exercise [41,42] which should increase intrinsic motivation. It is clear that variables, including type of activity, age, and gender, do influence PA motivation, so it is important to consider them when developing strategies to promote PA. In particular, selection of a type of PA that will satisfy individuals’ primary motives has great potential to enhance intrinsic motivation and encourage long-term participation, but there is little definitive research examining this issue.

Based on the low levels of PA among Malaysians, individualizing activities based on motives is critical in ensuring that PA recommendations and guidelines are met. High levels of motivation for any behavior are associated with greater effort and persistence. Most previous studies have only compared differences across age and gender [23,29,43]. Few studies have examined scores from measures of motivation using statistical techniques that are specifically designed to identify differences in motives between categories, such as types of PA. In this study our aim was to apply DFA to tease out meaningful distinctions between motives that are important for one gender or the other, for young adults compared to middle-aged adults, and, most importantly, for different types of PA. There is still a lack of research on how motivation differs between various demographics of the adult population, especially concerning the key issue of type of activity. Therefore, the aims of this study were to: a) examine significant differences in participation motivation across type of activity, age, and gender, and (b) investigate the motives for participation that best discriminated between gender, adult age categories and type of PA.

Moreover, the third aim of this study was to examine the construct validity of the PALMS based on the extent to which motive sub-scales discriminate between type of activity, age, and gender. Based on the constructs of the motives this study predicts that different motives will discriminate type of activity, age, and gender.

## Methods

### Ethics statement

The university Institute of Research Management and Monitoring and Sports Centre Research Committee approved the study. Participation in the study was voluntary and all participants provided written consent to participate in the study.

### Participants

A sample of 1,360 Malaysian volunteers (703 males, 657 females) who undertook regular PA (at least 150 minutes of moderate- to vigorous-intensity PA per week) during the last six months participated in this study. The participants were assigned to two age categories, according to Erikson’s stages of human development [44]: young adults aged 20 to 40 years (*n* = 763; *M* = 29.12; *SD* = 3.9) and middle-aged adults aged 41 to 64 years (*n* = 597; *M* = 54.21; *SD* = 4.32) (Table 1).

**Table 1:** **Descriptive statistics for the whole sample**

	***N***	**Minimum**	**Maximum**	***M***	***SD***
**Age (in years)**	1360	20	64	35.71	10.28
**Young adult**	763	20	40	28.8	6.49
**Middle-aged adult**	597	41	64	49.5	7.92
**Frequency of activity per week (number of times)**	1360	2	7	2.85	1.32
**Average duration of each session of activity (in minutes)**	1360	30	240	57.36	31.32
**Period of regular exercise (in months)**	1360	6	60	8.40	3.75

**Note:** *N* = sample size. *M* = Mean. *SD* = Standard deviation.

Participants took part in five types of activity, namely individual racing sports plus bowls (*n* = 272; bowling = 56, swimming = 68, track competitive running = 119, cycling = 39), team sports (*n* = 358; basketball = 72, football = 125, futsal = 116, volleyball = 45), racquet sports (*n* = 279; badminton = 159, tennis = 36, table tennis = 84), martial arts (*n* = 211; taekwando = 67, karate = 51, tai chi = 93) and exercise (*n* = 240; walking = 91, jogging = 61, dancing = 19, gym = 69). The participants did an average of 2.85 sessions of PA per week with each session lasting 57.38 ± 31.32 minutes. They had been participating in regular PA for 8.4 ± 3.8 months. Information on demographics was self-reported based on a demographics form.

### Measures

#### Demographics form

Participants reported key demographic variables, including gender, age, ethnicity, and PA. They also reported their primary PA, and the level, frequency, duration, and intensity of PA per week.

#### Motives for participation in PA

In the study, we administered the 40-item Physical Activity and Leisure Motivation Scale (PALMS) [45], which was designed to measure adult PA motivation. PALMS measures eight motives for participation in PA, namely mastery, enjoyment, psychological condition, physical condition, appearance, others’ expectations, affiliation, competition/ego, on a 5-point Likert scale ranging from 1 (*strongly disagree*) to 5 (*strongly agree*). The range of each PALMS sub-scale is 5 to 25 because each sub-scale has five items. Based on SDT, Morris and Rogers [45] categorized the eight motives as aspects of intrinsic motivation (mastery and enjoyment sub-scales) or extrinsic motivation (the other six sub-scales). They further classified the six extrinsic motives into two second-order factors, namely body-mind motives (physical condition, psychological condition, and appearance), and social motives (others’ expectations, affiliation, and competition-ego) on the basis of a second-order factor analysis of the eight motives [45]. The PALMS has been validated in previous studies. Chowdhury [29] distributed the PALMS to 202 volunteer participants, aged 18 to 71 years, from various organizations, clubs, and leisure centers in Australia. Results of a confirmatory factor analysis (CFA) indicated that the PALMS had a robust factor structure (*CMIN*/DF = 2.22; *NFI* = 0.95; *CFI* = 0.97; *RMSEA* = 0.078). Zach, Bar-Eli, Morris and Moore [46] validated the PALMS with 678 recreational exercise participants, aged 9 to 89 years, who exercised regularly in Israel. They reported that the PALMS demonstrated good internal consistency for each of the sub-scales, ranging from 0.63 to 0.96. In a study by Molanorouzi, Khoo and Morris [47], the PALMS was completed by 502 Malaysian participants, aged 17 to 67 years (*M* = 31.55; *SD* = 11.87). The hypothesized 8-factor model demonstrated a good fit with the data (*CMIN*/DF = 2.820, *NFI* = 0.90, *CFI* = 0.91, *RMSEA* = 0.06). Internal consistency for the PALMS sub-scales was sound, ranging from 0.78 to 0.82 and test-retest reliability for the questionnaire sub-scales was between 0.78 and 0.94 over a 4-week period [47].

### Procedure

Participants who exercised regularly (at least 150 minutes of moderate- to vigorous-intensity PA every week) in the last six months were recruited from various sports clubs and leisure centres from May to August 2013. Participants were given information sheets, signed consent forms, and completed the questionnaires in a private setting as they entered or left group activity, as they left the sports centers, or, occasionally, during breaks in their exercise routines. It was then explained to prospective participants that their participation was voluntary and that they could withdraw from the study at any point should they feel uncomfortable. Prospective participants who were willing to participate in the study were then told the nature and purpose of the study. They were also informed that there were no right or wrong answers and that their responses would be kept confidential. Participants were asked to fill in the questionnaire and return it to the researchers, who waited at the testing location. The 1,360 respondents took 10–12 minutes to complete the questionnaire.

### Data analysis

Data were screened for normality, outliers, and homogeneity. Participants with missing responses on one or more items (*n* = 23, 1.6% of sample) were excluded from subsequent analyses. First, descriptive statistics (frequencies, means, SDs) for the whole sample and each classification (type of activity, age, and gender) were calculated. Next, DFA was used to determine which motives for participation sub-scales could best distinguish between type of activity, age, and gender. Follow-up analysis of an independent t-test was also performed to identify significant between group differences in the variables assessed. Statistical significance was set at *p* < 0.05. SPSS version 21.0 was used for these analyses.

## Results

### Examination of motives by gender, age, and type of physical activity

#### Gender differences

DFA revealed a significant canonical function (*Wilks’ Lambda* = .619, *p* < .001), indicating that male and female groups could be effectively discriminated by the motivational sub-scales measured. DFA also identifies the extent to which group membership can be successfully predicted. In this analysis, 82% of the sample was correctly classified according to gender. Examination of the structure coefficients (Table 2), showed that competition/ego (0.97), appearance (0.77), physical condition (0.58), and mastery (0.48) contributed most to gender differences in PA participation, using a minimum discriminant function loading of ±0.30 [48].

**Table 2:** **Structure coefficients for gender and age canonical function**

**Sub-scale**	**Canonical structure coefficients**	
	**Gender function**	**Age function**
**Mastery**	.481	.868
**Enjoyment**	.274	.331
**Psychological condition**	-.159	-.830
**Physical condition**	-.579	-.803
**Appearance**	-.768	-.248
**Others’ expectation**	-.058	.021
**Affiliation**	-.098	.548
**Competition/Ego**	.972	.231

**Note:** A minimum canonical structure coefficient of ±0.30 considered significant [48].

Examination of group means and t-tests indicated that females reported higher motivation for appearance and physical condition than males, whereas males were more motivated by competition/ego and mastery than females (Table 3).

**Table 3:** **Motivation sub-scales according to age group and gender**

**Sub-scale**	**Age**		**Gender**	
	**20 to 40**	**41 to 64**	**Male**	**Female**
**Mastery**	20.90 ± 3.03*	17.71 ± 4.05*	20.61 ± 3.42*	18.30 ± 3.94*
**Enjoyment**	21.17 ± 2.92*	19.79 ± 3.42*	20.93 ± 3.03*	19.77 ± 3.37*
**Psychological condition**	20.08 ± 3.21*	21.64 ± 3.02*	19.89 ± 3.16*	20.80 ± 20.8*
**Physical condition**	20.42 ± 3.47*	20.22 ± 3.33*	19.86 ± 3.76*	20.84 ± 2.91*
**Appearance**	18.76 ± 4.25*	18.86 ± 3.93*	18.28 ± 4.27*	20.37 ± 3.86*
**Others’ expectation**	15.41 ± 4.54	16.19 ± 4.87	16.61 ± 4.59	14.84 ± 4.65
**Affiliation**	19.89 ± 3.37*	17.49 ± 4.34*	19.67 ± 3.58	17.95 ± 4.24
**Competition/Ego**	18.47 ± 4.43*	16.22 ± 4.79*	19.31 ± 4.15*	15.53 ± 4.53*

**Note:** Results are shown as Mean ± SD; *p < .05.

#### Age differences

DFA revealed a significant canonical function (*Wilks’ Lambda* = .590, *p* < .001), revealing that young and middle-aged adults could be effectively discriminated by the motivational sub-scales measured. In the case of young and middle-aged adults, 83% of the sample was correctly classified according to age category. Examination of the structure coefficients indicated that the mastery (0.87), psychological condition (0.83), physical condition (0.80), affiliation (0.55), and enjoyment (0.33) sub-scales contributed most to age differences (Table 2), using a minimum value of ±0.30 [48]. Examination of group means and t-tests indicated that young adults reported higher affiliation, mastery, and enjoyment associated with participation in PA than middle-aged adults, whereas middle-aged adults considered psychological condition and others’ expectations more important motives for participating in PA than young adults (Table 3).

#### Type of activity differences

To examine the extent to which the motives measured by the PALMS differentiated between participation in the five types of activity examined in the present study, we conducted five separate DFAs. In each case, we examined whether participants in the target type of PA could be discriminated from the rest of the sample combined on the basis of the eight motives measured by the PALMS.

DFA indicated a significant canonical function for team sport players (*Wilks’ Lambda* = .725, *p* < .001), revealing that team sport players and the rest of the sample could be effectively discriminated by the motivational sub-scales measured. In this case, 76% of the sample was correctly classified according to team sport category. Examination of the structure coefficients (Table 4), using a minimum discriminant function loading of ±0.30 [48], indicated that affiliation contributed most to the discriminant function, with mastery and physical condition also representing meaningful contributors (±0.30).

**Table 4:** **Structure coefficients for the five types of activity canonical function**

	**Canonical structure coefficients**				
	**Team sports function**	**Individual racing sports plus bowls function**	**Racquet sports function**	**Exercise function**	**Martial arts function**
**Mastery**	0.380	0.216	1.051	−0.562	0.301
**Enjoyment**	−0.085	1.333	−0.218	−0.282	−0.275
**Psychological condition**	−0.229	−0.246	0.218	1.225	0.314
**Physical condition**	0.319	−0.199	−0.195	0.341	0.296
**Appearance**	0.295	0.343	0.215	0.411	−0.221
**Others’ expectation**	−0.095	−0.143	0.226	0.218	−0.104
**Affiliation**	1.172	0.478	−0.299	−0.295	0.182
**Competition/Ego**	0.162	0.232	0.413	−0.426	1.330

**Note:** A minimum canonical structure coefficient of ±0.30 considered significant [48].

Examination of group means in Table 5 and a follow-up independent t-test showed that team sport participants reported higher motives for affiliation and mastery than the rest of the sample and a somewhat lower motive for physical condition.

**Table 5:** **Motivation sub-scales according to each type of activity vs rest of sample**

	**Type of activity**									
**Variables**	**T1**		**T2**		**T3**		**T4**		**T5**	
	**Team sports**	**Rest of sample**	**Individual racing sport plus bowls**	**Rest of sample**	**Racquet sports**	**Rest of sample**	**Exercise**	**Rest of sample**	**Martial art**	**Rest of sample**
**Mastery**	20.46 ± 3.37*	19.49 ± 5.11*	18.45 ± 3.27*	20.00 ± 4.72*	22.52 ± 2.53*	18.98 ± 3.82*	16.58 ± 3.62*	20.46 ± 4.65*	20.44 ± 2.56*	19.50 ± 3.78*
**Enjoyment**	19.27 ± 3.11	19.29 ± 4.25	21.49 ± 3.11*	18.73 ± 3.81*	19.72 ± 3.01	19.17 ± 4.70	17.60 ± 3.73*	19.70 ± 4.12*	18.35 ± 2.84*	19.52 ± 4.18*
**Psychological cond**	17.51 ± 3.07*	19.85 ± 4.09*	18.08 ± 2.80*	19.71 ± 5.14*	18.40 ± 3.12*	19.63 ± 3.89*	22.20 ± 3.16*	18.68 ± 3.90*	20.75 ± 2.37*	19.04 ± 3.86*
**Physical condition**	19.09 ± 3.47*	20.99 ± 3.87*	21.01 ± 3.24	20.51 ± 3.81	21.60 ± 3.28	20.39 ± 5.19	21.95 ± 3.42*	20.28 ± 3.88*	19.43 ± 2.17*	20.91 ± 3.78*
**Appearance**	19.80 ± 3.61*	18.39 ± 4.92*	16.91 ± 3.78*	19.11 ± 5.13*	19.15 ± 3.46	18.55 ± 4.56	21.61 ± 2.37*	17.94 ± 3.94*	15.91 ± 4.76*	19.36 ± 5.89*
**Others’ expectations**	17.64 ± 3.87*	15.09 ± 4.37*	14.34 ± 4.98*	15.91 ± 6.11*	15.74 ± 4.25	15.56 ± 3.94	14.83 ± 4.64	15.79 ± 5.17	15.46 ± 5.22	15.63 ± 4.36
**Affiliation**	21.78 ± 2.81*	17.38 ± 5.71*	16.60 ± 4.36*	18.68 ± 4.33*	18.23 ± 2.91*	18.27 ± 5.11*	15.30 ± 3.86*	19.00 ± 4.80*	19.42 ± 3.38	17.97 ± 3.81
**Competition/Ego**	19.63 ± 3.99	19.27 ± 4.62	18.36 ± 4.54*	19.59 ± 5.41*	20.27 ± 3.26*	19.11 ± 4.07*	15.32 ± 4.14*	20.35 ± 4.84*	23.15 ± 4.53*	18.39 ± 3.72*

**Note:** Results are shown as Mean ± SD; *p < .05.

For individual racing sport plus bowls participants, DFA reflected a significant canonical function (*Wilks’ Lambda* = .490, *p* < .001). This result indicated that individual racing sport plus bowls players and the rest of the sample could be effectively discriminated by the motivational sub-scales assessed. In this analysis, 91% of the sample was correctly classified according to individual racing sport plus bowls group. Examination of the structure coefficients (Table 4), using a minimum discriminant function loading of ±0.30 [48], revealed that enjoyment contributed most to the discriminant function, with appearance and affiliation sub-scales also representing meaningful contributors (±0.30). Examination of group means in Table 5 and a follow-up independent t-test showed that individual racing sport plus bowls participants were more motivated by enjoyment than the rest of the sample, but less motivated by appearance and affiliation.

Also, DFA showed a significant canonical function for racquet sport players (*Wilks’ Lambda* = .602, *p* < .001), indicating that racquet sport players and the rest of the sample could be effectively discriminated by the motivational sub-scales assessed. In this case, 90% of the sample was correctly classified according to whether they were from racquet sports or the rest of the sample. Examination of the structure coefficients (Table 4), using a minimum discriminant function loading of ±0.30 [48], revealed that mastery contributed most to the discriminant function, with the competition sub-scale also representing a meaningful contributor (±0.30). Examination of group means in Table 5 and a follow-up independent t-test showed that racquet sport participants reported a significantly higher motive for mastery than the rest of the sample and a higher motive for competition.

For exercisers, DFA indicated a significant canonical function (*Wilks’ Lambda* = .697, *p* < .001), showing that exercise and the rest of the sample could be effectively discriminated by the motivational sub-scales measured. In this analysis, 84% of the sample was correctly classified according to whether they were from the exercise group or the rest of the sample. Examination of the structure coefficients (Table 4), using a minimum discriminant function loading of ±0.30 [48], identified that psychological condition contributed most to the discriminant function, with the mastery, competition/ego, appearance, and physical condition sub-scale also representing meaningful contributors (±0.30). Examination of group means in Table 5 and a follow-up independent t-test showed that exercisers reported higher motives for psychological condition, appearance, and physical condition than the other participants in the study, but lower motives than the rest of the sample for mastery and competition/ego.

DFA indicated a significant canonical function for martial arts participants (*Wilks’ Lambda* = .595, *p* < .001). This finding indicated that martial arts participants and the rest of the sample could be effectively discriminated by the motivational sub-scales measured. In this case, 91% of the sample was correctly classified according to whether they participated in martial arts compared to the rest of the activities in the sample. Examination of the structure coefficients (Table 4), using a minimum discriminant function loading of ±0.30 [48], indicated that competition/ego contributed most to the discriminant function, with mastery and psychological condition also representing meaningful contributors (±0.30). Examination of group means in Table 5 and a follow-up independent t-test showed that martial arts participants reported higher motives for competition/ego, mastery, and psychological condition than the other participants in this sample.

## Discussion

In this study, we investigated differences in motives for PA across age, gender, and type of activity. Research on participation motivation suggests that there are systematic differences between participation motives related to demographic variables, such as age and gender [23,24]. The current findings showed that males were more motivated than females by mastery and competition/ego, whereas females were motivated more than males by appearance and physical condition. This is consistent with previous research, which found that men showed significantly higher intrinsic motivation based on desire to achieve mastery [23,49]. Also, this result supports past research, which revealed that men were more motived than women by competition and challenge [23,29,30]. Women, on the other hand, had higher scores then males for extrinsic motives related to physical attractiveness and appearance [23,29]. Indeed, the results of this study support previous studies and show that there are noteworthy differences on motivation sub-scales, especially in competition/ego and appearance sub-scales among males and females who participate in PA.

Some studies have demonstrated that decline in PA levels occurred more in females than males when males and females participated together in mixed-gender classes [31,32,50]. Based on the findings of the present study, health educators and health professionals might pay attention to different aspects of motivation in mixed sex classes because, among adults, male participants are likely to be more motivated by mastery and competition, whereas females are more motivated by appearance and body physical condition to improve and maintain PA levels. Additionally, health professionals might focus on providing opportunities for men to experience mastery (by learning new skills) and to compete (at a level where they experience success, because losing tends to reduce motivation) and, for females, health and exercise professionals should provide opportunities to enhance psychological well-being and to maintain or enhance their appearance.

This research distinguished different motives for participation in PA that young adults (aged 20 to 41) and middle-aged adults (aged 41 to 64) considered to be more important. This finding supports previous researchers [23,24], who found that older participants took part in PA because of more extrinsic motivation compared to younger participants.

Results of the current study also support past study, Trujillo, Brougham, and Walsh [33] suggested changes in motivation that occur as people age, based on the theories of Buhler [34,35]. They proposed that in later years, people are involved in the evaluation of their lives. Trujillo et al. further suggested that old age brings increasing concern with the deterioration of one’s physical health and the resulting increase in dependence on others. Exercise habits have a large impact on one’s physical health through the prevention of heart disease and strokes and maintaining physical strength [51]. PA also has a large impact on one’s psychological health by reducing stress and raising self-esteem, and possibly even maintaining cognitive abilities [52]. Thus, it is reasonable to expect that older adults would show more concern for physical and psychological health issues in making their PA decisions.

Researchers have reported that level of PA declined as age increased [10,31,50] and intrinsic motivation has been positively linked with exercise adherence. Indeed, participants with higher levels of intrinsic motivation have been shown to persist in activities for longer, and report higher levels of adherence [16-18,53]. The results of the present study suggest that health educators and professionals need to understand the importance of individual motivation sub-scales on measures like the PALMS to increase the likelihood of success in their physical activity interventions.

Five DFAs were conducted, one to compare each of the five PA types with the rest of the sample, to identify the motives that were particularly strong reasons for participation in that type of PA. We found that participants’ motives for participation in PA distinguished each of the five types of PA from the rest of the sample. The strongest discriminators were affiliation for team sports, enjoyment for individual racing sport plus bowls players, mastery for racquet sports, psychological condition for exercisers, and competition/ego for martial arts players.

The finding that team sport participants were most clearly discriminated from the rest of this sample by their motive for affiliation is consistent with the only previous study that examined team sports compared to a range of sport types similar to the ones examined here. In a sample of 2,601 Australian participants in sport and PA, Morris et al. [30] found that affiliation discriminated the team sports players from the other PA types most strongly on the PMQ. In a study that compared specific activities that represented different types of PA, Chowdhury [29] found that team sport participants in Australian football were discriminated most clearly from tennis, gym, yoga and tae kwon do participants by the affiliation motive on the PALMS. These findings represent the most robust discriminator of any type of PA found in this kind of research.

Individual racing sports plus bowls participants were discriminated from the rest of the sample most clearly by the motive of enjoyment. Comparisons of individual and team sports in previous research reflect similar patterns. For example, Frederick and Ryan [38] found that those who participated in individual racing sports plus bowls had higher interest/enjoyment and competence motivation than those who participated in team activities.

For racquet sport participants, mastery was the key discriminating motive. This result is not consistent with previous research [30]. Morris et al. reported that racquet sport competitors were discriminated by higher scores than the rest of the sample on the challenge or competition/ego sub-scale. Chowdhury [29] also found that the competition/ego motive was a strong discriminator of tennis players from participants in Australian football, gym, yoga, and tae kwon do. This inconsistency might relate to the level at which participants typically performed in these studies. In the study by Morris et al. [30] and the research conducted by Chowdhury [29] the tennis players were accessed from tennis clubs where most players were highly competitive. On the other hand, the tennis players in the present study were accessed at local tennis courts and might be described as recreational or developing participants. For sports performers who are learning the skills of an activity, it is likely that mastery is the primary motive and competition might become more prominent as their skills develop. For performers who have mastered the skills of an activity to a large degree and who play at a competitive level, perhaps in leagues, competition might replace mastery as the primary motive driving participation. The developing players in the present study reflected this pattern citing mastery as their primary motive on the PALMS, with competition as a secondary motive. In the two studies in which either racquet sports or tennis were examined among more competitive players, it is not surprising that competition/ego had replaced mastery as the primary motive for participation.

For exercisers the motive of psychological condition was the important discriminator. These findings are not entirely consistent with previous research by Morris et al. [30] who reported exercise participants were discriminated by higher scores on physical condition than participants in other activities. This might be because exercisers in this study (walking, jogging, dancing, and gym) were from different activities to those in the study by Morris et al. (aerobics, weight training). Many participants in activities like aerobics and weight training, especially those in the kinds of fitness centres where Morris et al. recruited participants were involved in relatively high-intensity exercise activities. Participants in the exercise category in the present study were in the main conducting lower intensity activities. It is possible that these participants focused more on general wellbeing with a psychological focus, whereas the main motive for participants in the Morris et al. study has sought out aerobics and weight training because they were more interested in improving their physical condition. Nonetheless, it should be noted that appearance and physical condition were also motives that discriminated the exercisers in the present study from the rest of the sample.

Finally, for martial arts participants in the present study, competition/ego was the main sub-scale compared to other types of activity. In the sample involved in the research by Morris et at. [30], using the PMQ, and in Chowdhury’s [29] PALMS study, it was found that psychological condition discriminated the martial arts from the other PA types most strongly. Again this could be related to varying skill levels in different studies. It is clear that participants in the present study were at a more developmental level than those in the previous research, where mastery is likely to be an important motive. It is also possible that differences in the specific activities between studies played a role. In the Morris et al. [30] study a substantial proportion of the martial arts participants came from taiji, an activity in which grace, beauty and mental relaxation play a significant part.

Comparison of motives between different types of activity participants suggests that people participating in several types of PA placed varied emphasis on different motives. These findings suggest that particular participation motives clearly distinguish between the different types of physical activities. It is understandable that people participate in different physical activities for different reasons [21,28,30,37]. This information gives practitioners an idea of the benefits likely to be derived from each activity. In this study, the sample was correctly classified in the five activity groups, suggesting that potential participants can be directed to types of activity that most closely match their motives, where they are likely to encounter others with similar aims because those activity types satisfy important motives for participation for those individuals [30,37]. Types of activity can also be promoted in different ways to take advantage of variations in primary motives for participation, and hopefully reduce the typically high drop-out rates from different types of activity, especially in the first few months after initiation of an activity. For example, team sport participants clearly valued affiliation more highly than other participants, whereas martial arts participants were more motivated by competition than other participants. Thus, for somebody who dislikes the discipline of exercising alone at unsocial hours, instead wanting to be with friends, participating in certain kinds of team sport might be a more appropriate form of PA than running, bicycle riding, or swimming alone. By having knowledge of the most important motives for participation in different types of PA, practitioners can direct people to activities that most suit them on the basis of their personal profile of motives for participation on measures like the PALMS. Given that the present study provides support for the major motives that characterize different types of activity [28,30,37], the results suggest that people could be matched to a type of activity based on their principal motives. This would leave choice of specific activity, based on access, cost, culture, preferences and the like, but reduce the risk of rapid dropout by targeting a type of activity that matches primary motives.

A secondary aim of this study was to examine the construct validity of the PALMS as a measure of motives for participation in PA. Construct validity provides support to the validity of measures to the extent that predictions made from the construct are supported by studies testing those predictions. In this study, we examined the construct validity of the PALMS by testing the prediction that different motives would predict participation in different types of PA, age, and gender based on theory and previous research. The results of the present study supported the construct validity of PALMS by showing that the motives for participation in PA were different for females from males. Consistent with theory and research males were motivated more by competition and mastery, whereas females were motivated more by appearance and psychological condition. Younger adults were motivated primarily by mastery of the activity, whereas middle-aged adults were primarily motivated by psychological condition, as predicted by theory and previous research. The most specific predictions were made for the motives that discriminated each type of PA from the rest of the sample. In a number of types of PA the primary motives were consistent with previous research. In two cases, namely racquet sports and martial arts, the predictions were reversed compared with previous research, but were consistent with the specific activities and the level of participation in those activities observed in the present study. Therefore, this study provided support for a number of predictions, while raising some direction for further research that supported the construct validity of the PALMS.

### Limitations

This study had limitations in its conception and conduct. A number of issues have been raised in discussion of the findings that relate to the level at which participants in the present study participated in the activities to which they attributed their motives. While this study targeted certain types of activity, otherwise, the study employed a convenience sample, which was not controlled to monitor level of participation. Some trends that were observed could be attributable to the level at which participants took part in the activities. We consider that the sample in the present study was less competitive and more recreational than in previous studies. In addition, the specific activities that emerged from this convenience sample could have influenced the activities that were found to be the strongest discriminators. In further research, these issues should be addressed by more systematic sampling of specific activities and of the level at which participants are involved.

The present study focused on the relationship between motives and types of PA, as well as gender and age, using DFA to identify the motives that discriminated each type of activity from the rest of the sample. This approach has only been used in two previous studies to our knowledge. In the study by Morris et al. [30], a large sample was recruited, but a different questionnaire, the PMQ, was employed to measure motives for participation in PA. Although the PMQ has some similarity to the PALMS, differences between these measures are evident. This limits the confidence with which we can compare these two studies. The study conducted by Chowdhury [29] used the same questionnaire that was employed in the present study, but that study examined five specific activities in a much smaller sample than the present study. Thus, there is a need to replicate the present study with large samples of carefully selected participants and activities in a range of contexts both in Malaysia and in other countries. Discussion of the outcomes of this research suggest some directions in which to extend the present research. It has been suggested here that the level at which individuals participate, such as recreational, club, state, or national level, might influence motives in a way that is independent of the types of activity. This can be examined by systematically sampling participants from each activity to represent different levels of participation. We also argued that differences might exist between activities that have been categorized as belonging to the same type of activity in this kind of research. An example is classifying aerobics and weight training in the same category as walking and dancing. Although there are many classification systems for PA, the great diversity of PA means that, presently, no classification satisfactorily encompasses all physical activities. Further research should be conducted to refine the activities that can be confidently grouped together on the basis of motives for participation. Such research might employ cluster analysis to group activities together on the basis of the motives that characterize them.

## Conclusion

The findings of the present study illustrate the importance of age, gender and, in particular, type of activity when investigating PA motivation. This study was an important first step in understanding differences in motives for participation in various types of PA and associations between motives for participation in PA and PA behaviour across the adult lifespan. Most importantly, the results of this study highlighted the message that understanding strong participation motives across type of activity, age, and gender may be effective in promoting PA in adults.

## Abbreviations

PA: Physical Activity
PALMS: Physical Activity and Leisure Motivation Scale
SDT: Self-determination Theory
DFA: Discriminant Function Analysis
PMQ: Participation Motivation Questionnaire
